# Polynucleotides and Polydeoxyribonucleotides for Skin Rejuvenation, Postoperative Scar Prevention, and Wound Healing: A Systematic Review of Randomized Clinical Trials

**DOI:** 10.7759/cureus.112403

**Published:** 2026-07-10

**Authors:** Ahmed M Alhussain, Sarah Turki M. Albusayys, Mohammed A Alfhadi, Saleh A Bu Mozah, Shahad Saad S AlShammari, Husam Abdullah M Alanazi, Sara Adil Alqazanli, Ali Nouruldeen Alabdullah, Noorah M. ALrashidi, Abdulrahman A Almazrou, Abdulelah F Alshehri

**Affiliations:** 1 College of Medicine, Almaarefa University, Riyadh, SAU; 2 College of Medicine, Najran University, Najran, SAU; 3 College of Medicine, King Faisal University, Al-Ahsa, SAU; 4 Department of Ophthalmology, Imam Mohammad ibn Saud Islamic University, Riyadh, SAU; 5 Clinical Research, Independent Research, Riyadh, SAU

**Keywords:** polydeoxyribonucleotide, polynucleotide, scar prevention, skin rejuvenation, wound healing

## Abstract

Polynucleotides (PNs) and polydeoxyribonucleotides (PDRNs) are increasingly used in regenerative dermatology; however, randomized evidence across skin rejuvenation, scar prevention, and wound healing has not been comprehensively synthesized. This systematic review evaluated randomized clinical trials investigating PN- and PDRN-based interventions in these indications. PubMed, Embase, and the Cochrane Central Register of Controlled Trials (CENTRAL) were searched through January 2026. Seven randomized trials involving 183 participants met the inclusion criteria. Four studies evaluated skin rejuvenation, one postoperative scar prevention, and two wound-healing applications. Across studies, PN and PDRN therapies improved wrinkle-related outcomes, scar quality, wound-healing metrics, and patient-reported satisfaction. The most consistent benefits were observed in wound-healing settings, where PDRN accelerated re-epithelialization and shortened healing time compared with controls. No serious treatment-related adverse events were reported. Although findings support the regenerative potential of PN- and PDRN-based therapies across multiple dermatologic applications, the evidence base remains limited by small sample sizes, heterogeneous treatment protocols, and variable outcome measures. Larger, adequately powered randomized trials are needed to establish optimal clinical indications and treatment strategies.

## Introduction and background

Skin aging, postoperative scar formation, and impaired wound healing are common clinical conditions that share several underlying biological mechanisms, including extracellular matrix degradation, altered collagen remodeling, fibroblast dysfunction, chronic inflammation, and dysregulated tissue repair. These processes contribute to functional impairment, aesthetic concerns, reduced quality of life, and substantial healthcare burden worldwide [1]. As demand for aesthetic procedures continues to rise and the burden of surgical scars and chronic wounds remains significant, interest has increasingly shifted toward regenerative therapies that restore tissue structure and function through biologically active mechanisms rather than solely providing symptomatic or cosmetic improvement [2]. Current treatment strategies include topical agents, injectable fillers, laser therapies, radiofrequency devices, and surgical interventions. Although these approaches may improve clinical appearance, their effects are often variable and may not directly target the molecular pathways involved in tissue regeneration and long-term tissue remodeling. Consequently, regenerative dermatology has emerged as an evolving field focused on therapies that enhance endogenous repair processes and restore tissue function at the cellular level [3,4].

Polynucleotides (PNs) and polydeoxyribonucleotides (PDRNs) have attracted increasing attention as regenerative therapies in dermatology. These highly purified DNA-derived compounds exhibit biological activities that support tissue repair and regeneration. Experimental and clinical studies have demonstrated that PDRN may enhance cellular proliferation, extracellular matrix remodeling, and tissue regeneration. Mechanistic investigations have further suggested that PDRN promotes tissue repair through the activation of adenosine A2A receptors, leading to enhanced angiogenesis, the modulation of inflammatory responses, the stimulation of fibroblast activity, and increased extracellular matrix synthesis [5]. Clinical investigations have increasingly evaluated PN- and PDRN-based therapies across multiple dermatologic indications. Recent clinical studies have reported favorable outcomes of PN-based therapies for postoperative scar management following thyroidectomy, supporting their potential role in modulating wound remodeling and scar quality [6]. In aesthetic medicine, PN-based interventions have demonstrated beneficial effects on wrinkle severity, skin quality, and patient-reported cosmetic outcomes [7]. Similarly, PDRN has shown promising results in wound-healing applications, including pressure ulcers and skin-graft donor sites, where accelerated re-epithelialization and enhanced tissue repair have been reported [8,9]. Despite growing clinical use, the available evidence remains fragmented. Existing studies vary considerably with respect to formulations, administration techniques, comparator interventions, outcome measures, and follow-up duration. In addition, skin rejuvenation, postoperative scar prevention, and wound healing are often investigated separately despite sharing overlapping biological pathways that may respond to similar regenerative mechanisms. Consequently, the overall efficacy and safety of PN- and PDRN-based therapies across the spectrum of dermatologic tissue repair remain incompletely characterized. Therefore, this systematic review aimed to synthesize evidence from randomized clinical trials evaluating the efficacy and safety of PN- and PDRN-based interventions for skin rejuvenation, postoperative scar prevention, and wound healing.

## Review

Materials and methods

Protocol and Reporting Standards

This systematic review was conducted in accordance with the Preferred Reporting Items for Systematic Reviews and Meta-Analyses (PRISMA) 2020 statement [10]. This systematic review aimed to evaluate the clinical efficacy and safety of PN- and PDRN-based interventions in skin rejuvenation, postoperative scar prevention, and wound healing. Given the substantial clinical and methodological heterogeneity among the included studies, a quantitative meta-analysis was not performed. Included trials differed with respect to patient populations, intervention formulations, treatment delivery methods, clinical indications, outcome measures, and follow-up durations. Consequently, statistical pooling was considered inappropriate, and findings were synthesized using a structured narrative approach. The review protocol was prospectively registered in the International Prospective Register of Systematic Reviews (PROSPERO) (registration number: CRD420261418096).

Literature Search Strategy

A comprehensive electronic literature search was conducted in PubMed, Embase, and the Cochrane Central Register of Controlled Trials (CENTRAL) from database inception through January 2026. Additional studies were identified through manual screening of reference lists from eligible articles. Search results were exported and managed using EndNote 20 (Clarivate Analytics, Philadelphia, Pennsylvania, United States). The search strategy combined controlled vocabulary and free-text terms related to polynucleotides, polydeoxyribonucleotides, skin rejuvenation, scar prevention, wound healing, aesthetic dermatology, and randomized clinical trials. Boolean operators (AND, OR) were used to combine search concepts.

Eligibility Criteria

Studies were considered eligible if they met the following criteria: (1) randomized clinical trials, including parallel-group, split-face, double-blinded, or patient-blinded randomized designs; (2) evaluated PN- or PDRN-based interventions; (3) investigated applications related to skin rejuvenation, postoperative scar prevention, or wound healing; (4) included human participants; and (5) reported extractable efficacy or safety outcomes. Studies were excluded if they were non-randomized studies, observational studies, case reports, case series, review articles, expert consensus statements, protocol publications, animal studies, in vitro investigations, or studies lacking extractable outcome data.

Study Selection

Two reviewers independently screened titles and abstracts for eligibility. Potentially relevant studies subsequently underwent full-text assessment. Disagreements during screening and eligibility assessment were resolved through discussion and consensus, with consultation of a third reviewer when necessary.

Data Extraction

Data extraction was performed independently by two reviewers using a standardized form developed a priori. Extracted variables included study characteristics, participant demographics, intervention and comparator details, sample size, treatment protocols, follow-up duration, efficacy outcomes, safety outcomes, and adverse events. Extracted efficacy outcomes included wrinkle severity measures, scar assessment scales, wound-healing metrics, patient satisfaction measures, and treatment-related adverse events. For split-face studies, data from treatment and comparator sides were extracted separately where applicable.

Risk of Bias Assessment

Methodological quality was assessed using the Cochrane Risk of Bias 2 (RoB 2) tool. The following domains were evaluated: bias arising from the randomization process, deviations from intended interventions, missing outcome data, outcome measurement, and selection of reported results. Two reviewers independently assessed each study, and disagreements were resolved through discussion and consensus. Overall risk of bias was categorized as low risk, some concerns, or high risk according to RoB 2 guidance.

Data Synthesis

Given the heterogeneity of study populations, intervention protocols, outcome measures, and follow-up durations, quantitative synthesis was not undertaken. Findings were synthesized narratively and organized according to predefined clinical domains, including skin rejuvenation, postoperative scar prevention, and wound healing. Risk of bias assessments were incorporated into the interpretation of the evidence.

Results

Study Selection

**Figure 1:**
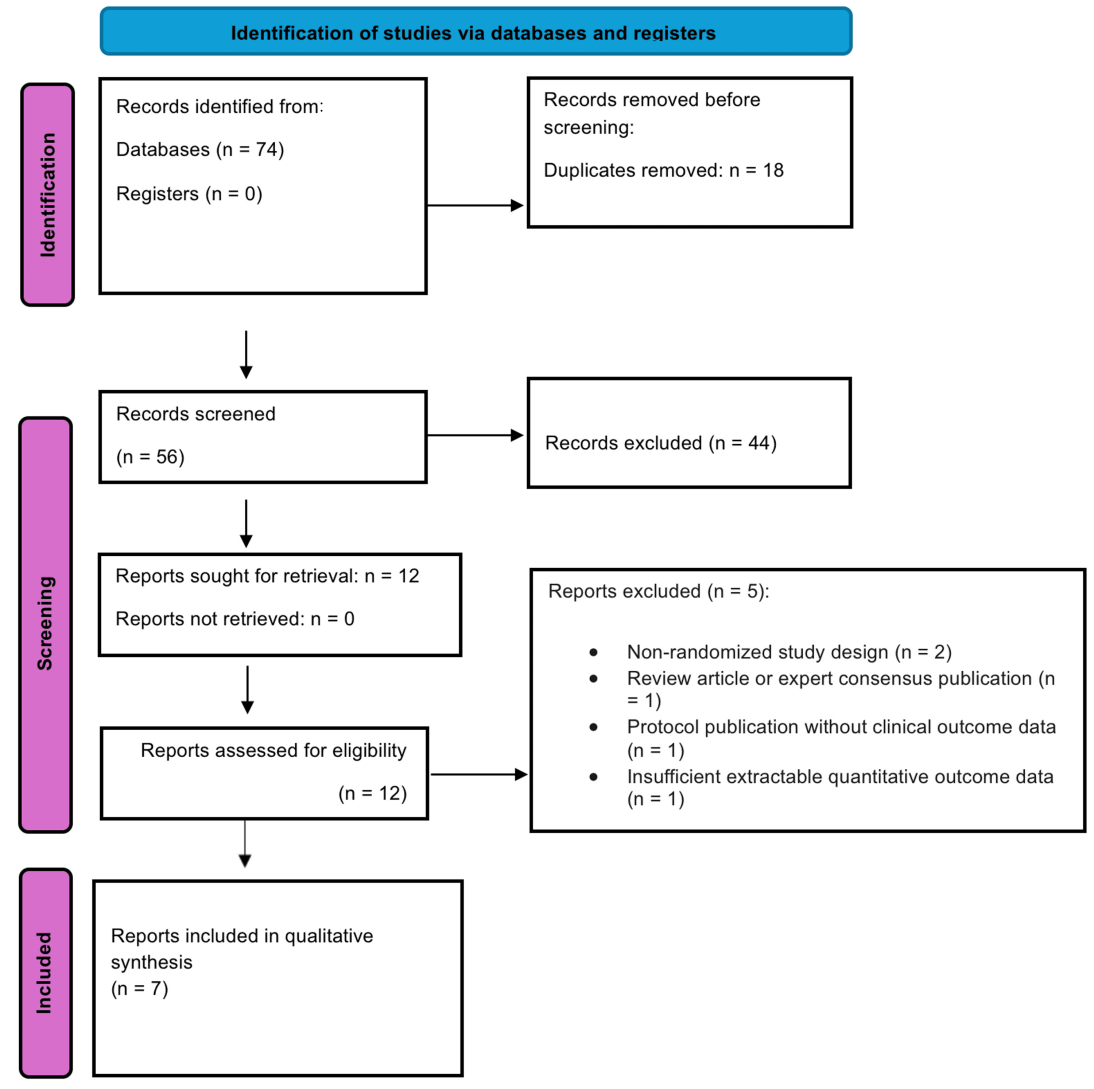
PRISMA flow diagram of the study selection process PRISMA flow diagram illustrating the process of study identification, duplicate removal, screening, eligibility assessment, and inclusion in the qualitative synthesis, adapted from the PRISMA 2020 statement [10]. PRISMA: Preferred Reporting Items for Systematic Reviews and Meta-Analyses

A total of 74 records were identified through database searching. Following the removal of 18 duplicate records, 56 records remained for title and abstract screening. Of these, 44 records were excluded because they did not meet the predefined eligibility criteria. Twelve full-text articles were subsequently retrieved and assessed for eligibility. Following full-text review, five articles were excluded for the following reasons: non-randomized study design (n=2), review article or expert consensus publication (n=1), protocol publication without clinical outcome data (n=1), and insufficient extractable quantitative outcome data (n=1). The remaining seven randomized clinical trials fulfilled all eligibility requirements and were included in the qualitative synthesis. These studies formed the evidence base for the evaluation of PN and PDRN applications in aesthetic skin rejuvenation, postoperative scar prevention, and wound repair. The study selection process is summarized in Figure 1.

Study Characteristics

Seven randomized clinical trials involving a total of 183 participants were included in the qualitative synthesis. The evidence base comprised both parallel-group randomized controlled trials and randomized split-face clinical trials. Four studies evaluated PN-based interventions, whereas three studies investigated PDRN-based therapies across skin rejuvenation, scar management, and wound-healing indications.

The included studies evaluated three principal dermatologic applications of PNs and PDRNs: aesthetic skin rejuvenation, postoperative scar prevention, and wound healing.

Four studies investigated aesthetic skin rejuvenation, one evaluated postoperative scar prevention following thyroidectomy, and two assessed wound-healing interventions in pressure ulcers and skin-graft donor sites.

The evidence base comprised randomized controlled trials, randomized split-face trials, and double-blinded controlled studies conducted across Asian and European populations. Publication years ranged from 2004 to 2025, reflecting both the early clinical investigation of PDRN in wound repair and the more recent expansion of PN-based therapies into aesthetic dermatology. Sample sizes ranged from 10 to 42 analyzed participants, with follow-up durations varying from two weeks to six months according to the clinical indication and study design. Split-face designs were employed in three of the four aesthetic rejuvenation studies (Yogya et al. [7]; Hong et al. [11]; Jeon et al. [12]), enabling within-subject comparisons and reducing confounding from interindividual variability, whereas parallel-group designs were used across the remaining studies.

Outcome assessment incorporated validated clinical grading scales, objective imaging modalities, wound-healing metrics, and patient-reported outcome measures. Detailed study characteristics and key study outcomes are summarized in Table 1.

**Table 1:** Characteristics of the included randomized controlled trials Summary of the study characteristics including country, study setting, participant characteristics, intervention, comparator, follow-up duration, and primary outcome measures. PN: polynucleotide; PDRN: polydeoxyribonucleotide; RFMN: noninsulated radiofrequency microneedling; GAIS: Global Aesthetic Improvement Scale; VAS: Visual Analog Scale; CFGS: Crow's Feet Grading Scale; VSS: Vancouver Scar Scale; SHI: Skin Hyperpigmentation Index; PRP: platelet-rich plasma; PUSH: Pressure Ulcer Scale for Healing

Study	Clinical domain	Population (n)	Intervention	Comparator	Follow-up	Main outcomes	Key findings
Yogya et al., 2022 [7]	Aesthetic skin rejuvenation	Women with periorbital wrinkles (n=29)	RFMN + topical PN	RFMN + saline	6 months	Wrinkle indentation, wrinkle depth, patient satisfaction	PN accelerated wrinkle improvement and produced greater early improvement in indentation outcomes
Hong et al., 2025 [11]	Aesthetic skin rejuvenation and treatment delivery	Adults seeking facial rejuvenation (n=10)	Needle-free PN delivery (CureJet)	Conventional PN injection	2 weeks	Pain, GAIS, satisfaction, wrinkle and pore indices	Needle-free delivery reduced pain and improved satisfaction while maintaining efficacy
Jeon et al., 2026 [12]	Aesthetic skin rejuvenation and formulation optimization	Patients with periorbital wrinkles (n=15)	Ionization-adjusted PN filler	Conventional PN filler	12 weeks	VAS pain, CFGS, GAIS, local reactions	Comparable aesthetic efficacy with significantly lower injection pain and fewer local reactions
Kim et al., 2018 [13]	Postoperative scar prevention	Thyroidectomy patients (n=42)	Fractional laser + PN	Fractional laser + saline	16 weeks	VSS, scar height, erythema, pigmentation	PN significantly improved scar quality, reduced scar height, and improved scar appearance
Vera et al., 2025 [14]	Aesthetic skin rejuvenation	Women with wrinkles and hyperpigmentation (n=24)	Microneedling + PDRN	Microneedling + PRP	6 weeks	Lemperle score, SHI	Both treatments improved wrinkle severity and hyperpigmentation; PDRN demonstrated greater wrinkle reduction, whereas pigmentation outcomes were comparable
Kim et al., 2014 [8]	Chronic wound healing	Pressure ulcer patients (n=23 analyzed)	Standard care + PDRN	Standard care	4 weeks	Wound area, PUSH score	PDRN significantly reduced wound surface area and improved PUSH scores throughout follow-up
Valdatta et al., 2004 [9]	Acute wound healing	Skin graft donor-site patients (n=40)	PDRN ointment	Conventional dressing	Until healing	Re-epithelialization, healing time, infection	PDRN accelerated healing, enhanced re-epithelialization, and reduced infection rates

Aesthetic Skin Rejuvenation Outcomes

Four randomized trials evaluated the use of PN- and PDRN-based interventions for aesthetic skin rejuvenation, encompassing the treatment of periorbital wrinkles, facial photoaging, hyperpigmentation, and patient-reported cosmetic outcomes. Across studies, nucleotide-based regenerative therapies demonstrated consistent improvements in wrinkle-related parameters while maintaining favorable tolerability profiles.

Yogya et al. [7] evaluated noninsulated radiofrequency microneedling (RFMN) alone versus RFMN combined with topical PN in a randomized split-face trial involving 29 women with Fitzpatrick skin types III to V. Objective assessment using Antera 3D imaging demonstrated that wrinkle indentation began to improve earlier on the PN-treated side, with a statistically significant between-group difference evident at the two-month follow-up (p<0.05; p=0.006). Steady improvement was observed on both the treatment and control sides through the six-month follow-up (p<0.05 and p=0.041, respectively). However, no statistically significant differences in maximum wrinkle depth were observed between the PN and control sides at any follow-up time point (p>0.05), indicating that the adjunctive benefit of PN was specific to wrinkle indentation rather than depth. At the six-month follow-up, 82% of subjects on both sides reported more than 25% self-assessed improvement, and 51% reported improvement exceeding 50%.

Vera et al. [14] conducted a randomized controlled trial in 24 women aged 30-50 years comparing microneedling combined with PDRN salmon 3% (Group 1; n=12) versus microneedling combined with platelet-rich plasma (Group 2; n=12). Using the Lemperle scoring system, both groups demonstrated statistically significant within-group reductions in wrinkle severity at six weeks. In Group 1, the mean Lemperle score decreased from 2.33±0.49 at baseline to 1.00±0.43 at follow-up (p=0.001), representing a 57% reduction. In Group 2, the mean score decreased from 2.33±0.49 to 1.50±0.52 (p=0.002). Between-group comparison demonstrated a statistically significant difference in favor of the PDRN group (p=0.021), indicating that microneedling combined with PDRN produced superior wrinkle reduction compared with microneedling combined with PRP at the six-week assessment.

In the same study (Vera et al. [14]), facial hyperpigmentation was assessed using the Skin Hyperpigmentation Index (SHI). Statistically significant within-group reductions in SHI scores were observed in both groups at six weeks. In Group 1, the mean SHI score decreased from 2.83±0.15 to 2.62±0.17 (p=0.001) and in Group 2 from 2.76±0.17 to 2.60±0.16 (p=0.001). However, no statistically significant between-group difference was identified (p=0.758), indicating that microneedling combined with PDRN and microneedling combined with PRP produced comparable reductions in facial hyperpigmentation at this time point.

Two studies evaluated alternative PN delivery strategies aimed at reducing injection-related pain while preserving aesthetic efficacy. Hong et al. [11] conducted a randomized split-face trial in 10 Korean adults comparing conventional intradermal PN needle injection with needle-free jet injection using the CureJet system. Pain intensity was significantly lower in the needle-free jet injection group (VAS 2.9±1.52) compared with the conventional needle injection group (VAS 5.4±1.42; p=0.017). Global Aesthetic Improvement Scale (GAIS) scores assessed by two independent dermatologists were significantly higher in the CureJet-treated areas (3.85±0.63) compared with the manually injected areas (3.60±0.52; p=0.037). Overall patient satisfaction scores were also significantly greater following needle-free injection (4.5±0.53 versus 3.9±0.73; p=0.019). Objective skin biophysical assessment using the Mark-Vu system demonstrated statistically significant improvements in pore index (p=0.009) and wrinkle index (p<0.001) in the needle-free jet injection areas at two weeks, whereas no significant pre- to post-treatment differences were observed in the conventional needle injection areas for either parameter.

Jeon et al. [12] evaluated an ionization-adjusted PN filler with modified pH and ionic strength in a randomized patient-blinded split-face trial involving 15 participants. Across 60 injection procedures, the mean VAS pain score was significantly lower for the ionization-adjusted formulation (3.39±1.81) compared with the conventional PN filler (5.04±2.19; p<0.005), with the ionization-adjusted filler producing lower pain scores in 48 of 60 procedures (80%). Post-procedural erythema was observed in 40% of ionization-adjusted PN filler procedures compared with 60% of conventional PN filler procedures (p=0.0075), and embossing occurred in 13.3% versus 56.7% of procedures, respectively (p<0.0001). Both formulations demonstrated statistically significant improvements in Crow's Feet Grading Scale (CFGS) scores from baseline at two weeks (p<0.001 for both), with improvements maintained through the 12-week follow-up, and no statistically significant between-group difference in CFGS or GAIS scores was identified at any assessment time point, confirming non-inferior aesthetic efficacy for the ionization-adjusted formulation. Across all four aesthetic rejuvenation studies, local adverse reactions were predominantly mild and transient. No serious adverse events, including infection, persistent erythema, pigmentary alteration, or scarring, were reported in any study.

Postoperative Scar Modulation

One randomized double-blinded controlled trial (Kim et al. [13]) evaluated PN treatment for postoperative scar prevention following open total thyroidectomy in 42 patients with thyroid carcinoma (22 in the PN group and 20 in the control group). All patients underwent a single combined session of ablative and non-ablative fractional laser treatment, with concomitant injection of either PN or normal saline, followed by two additional injection sessions at two-week intervals. Outcomes were assessed at baseline and at two, four, eight, and 16 weeks after treatment initiation.

Primary outcome assessment using the Vancouver Scar Scale (VSS) demonstrated significantly greater improvement in the PN group over the 16-week follow-up period. The mean VSS scores decreased from 6.05±0.52 at baseline to 2.09±0.47 at 16 weeks in the PN group, compared with a reduction from 6.25±0.59 to 4.01±0.55 in the control group (p<0.05). The PN group achieved greater than 50% reduction in VSS score, satisfying the predefined criterion for treatment success.

Analysis of individual VSS subscales revealed that pigmentation scores decreased significantly in the PN group from 1.64±0.10 at baseline to 0.77±0.13 at 16 weeks (p<0.05) and vascularity scores decreased from 1.41±0.14 to 0.32±0.12 (p<0.05). Pliability scores decreased from 1.73±0.16 to 0.59±0.16, and height scores decreased from 1.27±0.22 to 0.41±0.16; however, neither reached statistical significance (p=0.07 and p=0.06, respectively).

Three-dimensional image analysis demonstrated that the mean scar height was significantly lower in the PN group at the 16-week assessment (0.23±0.03) compared with the control group (0.29±0.03; p<0.05). Within the PN group, the mean scar height decreased from 0.46±0.06 at baseline to 0.23±0.03 at 16 weeks, whereas in the control group, it decreased from 0.38±0.04 to 0.29±0.03. The rate of improvement in scar height following the initial combined laser and injection session was significantly more pronounced in the PN group (p<0.05).

Spectrophotometric assessment demonstrated a significant decrease in the Erythema Index (EI) in the PN group compared with the control group after 12 weeks (PN group: 381.09±10.68 to 367.47±11.48; control group: 400.03±21.69 to 400.05±23.71; p<0.05). Although both groups demonstrated improvement in the Melanin Index (MI), the reduction was less prominent in the PN group (PN group: 148.06±10.04 to 134.76±8.57; control group: 147.56±9.79 to 113.41±9.37; p<0.05).

Patient scar perception scores improved from 2.86±0.18 to 3.27±0.18 in the PN group, compared with minimal change from 2.91±0.16 to 2.90±0.23 in the control group, though between-group differences did not reach statistical significance (p=0.23). No patient in either group developed hypertrophic scar or keloid formation during the 16-week follow-up. No adverse events, including post-inflammatory hyperpigmentation, scarring, or infection, were observed.

Cutaneous Wound Repair

Two randomized studies evaluated the effects of PDRN-based therapies on wound-healing outcomes in distinct clinical settings: pressure ulcers and autologous split-thickness skin-graft donor sites.

Kim et al. [8] conducted a randomized controlled trial in 23 analyzed patients with stage 2-4 pressure ulcers (experimental group n=11; control group n=12). The experimental group received PDRN (Placentex Integro) administered intramuscularly five times per week for two weeks, combined with perilesional infiltration twice per week for four weeks, in addition to standard wound care. The control group received standard wound care alone.

Wound surface area, measured using VISITRAK Digital, demonstrated progressive and statistically significant reductions in the PDRN group at all weekly assessment time points compared with controls. At week 1, the median wound surface area was 5.30 cm² (range: 2.50-8.70) in the experimental group versus 9.52 cm² (range: 4.80-17.00) in the control group (p=0.007). At week 2, values were 2.30 cm² (range: 0.30-5.90) versus 6.45 cm² (range: 3.00-16.00; p=0.001), at week 3, 1.30 cm² (range: 0.30-5.00) versus 5.70 cm² (range: 2.00-16.00; p=0.001), and at week 4, 0.90 cm² (range: 0.20-4.50) versus 4.25 cm² (range: 1.80-14.00; p<0.001).

Secondary outcome assessment using the Pressure Ulcer Scale for Healing (PUSH Tool 3.0) similarly demonstrated significantly lower scores in the experimental group at each time point. At week 1, the median PUSH scores were 11 (range: 8-14) versus 13 (range: 10-15; p=0.008), at week 2, 10 (range: 6-13) versus 12.5 (range: 10-14; p=0.002); at week 3, 8 (range: 5-11) versus 11 (range: 8-14; p=0.001), and at week 4, 4 (range: 2-10) versus 11 (range: 6-13; p<0.001). Hematological examinations conducted at week 4 revealed no abnormal findings in either group, and no adverse effects were reported throughout the treatment period.

Valdatta et al. [9] conducted a randomized pilot study in 40 patients undergoing split-thickness skin-graft harvesting, comparing topical PDRN ointment (n=20) with conventional non-adherent gauze dressing (n=20). Re-epithelialization was assessed at days 7 and 15 by photographic analysis.

At day 7, the mean percentage of re-epithelialization was 46.75% (range: 0-90%) in the PDRN group compared with 19.25% (range: 0-55%) in the control group (p<0.01). At day 15, the mean re-epithelialization reached 98.5% (range: 80-100%) in the PDRN group compared with 53.5% (range: 0-95%) in the control group (p<0.01). Complete re-epithelialization occurred at a mean of 12.5 days (range: 9-20) in the PDRN group compared with 24.45 days (range: 17-41) in the control group, representing an approximate 50% reduction in healing time.

Infection outcomes strongly favored PDRN treatment: no infections were recorded in the PDRN group throughout the study period, whereas nine infections occurred in the control group, representing a 45% infection rate. Subjective symptom assessment demonstrated that PDRN-treated patients were nearly completely asymptomatic, with only four cases of moderate symptoms reported, while the control group included four cases of serious symptoms and nine cases of marked symptoms, largely attributable to infection-related complications. No adverse reactions to the PDRN ointment or its excipients were reported.

Risk of Bias Assessment

**Figure 2:**
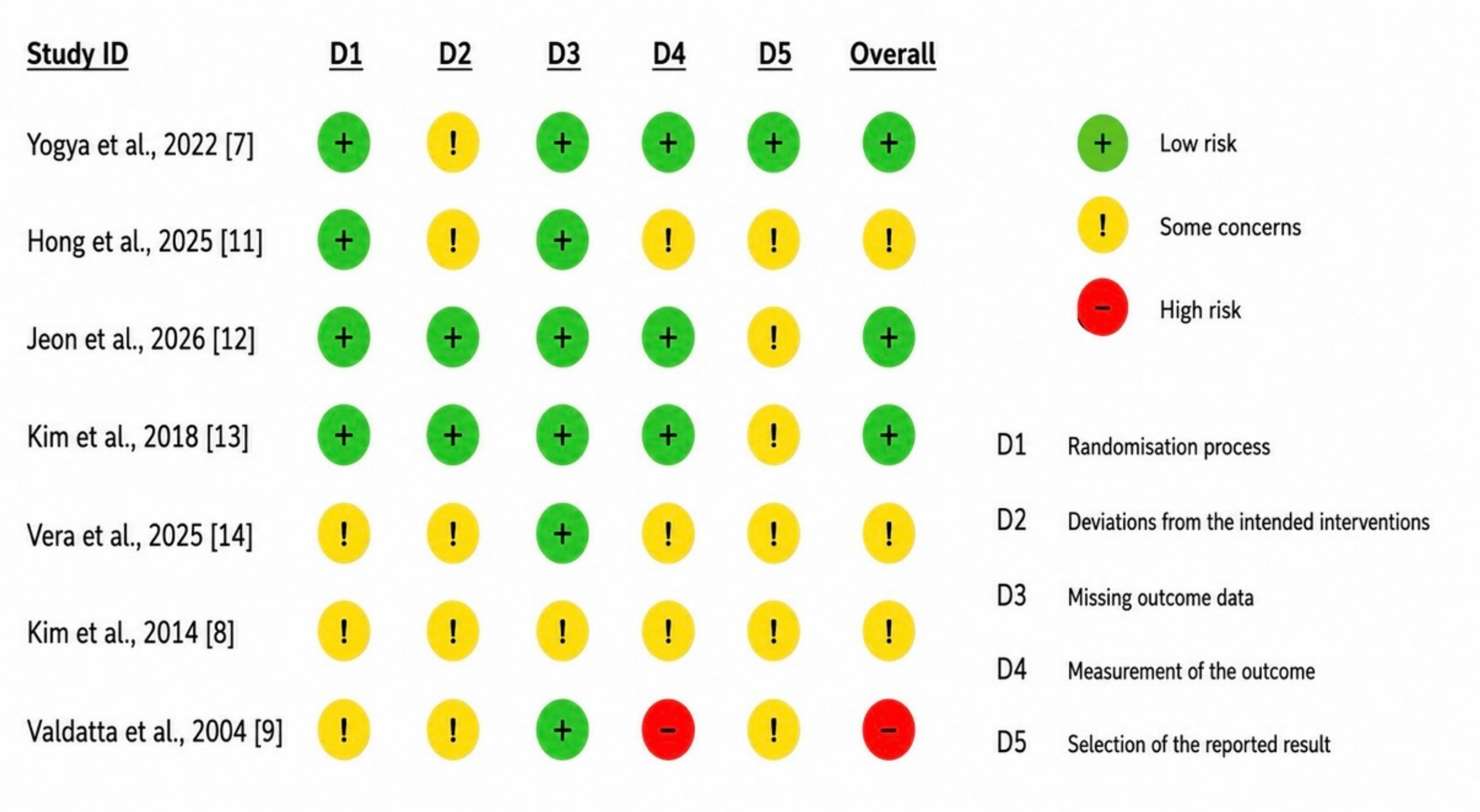
Risk of bias assessment across the included randomized controlled trials

Risk of bias was assessed using the RoB 2 tool across five domains. Overall, three studies were judged as having a low risk of bias, three studies were judged as having some concerns, and one study was judged as having a high risk of bias. Low-risk judgments were assigned to Yogya et al. [7], Jeon et al. [12], and Kim et al. [13]. Studies judged as having some concerns included Hong et al. [11], Vera et al. [14], and Kim et al. [8], primarily because of insufficient reporting of allocation concealment, deviations from intended interventions, or incomplete reporting of prespecified analyses. Valdatta et al. [9] was judged to have a high overall risk of bias due to the absence of blinding, potential bias in outcome measurement, and manufacturer sponsorship. Detailed domain-level assessments are presented in Figure 2.

Discussion

The present systematic review synthesized randomized evidence evaluating the clinical utility of PN- and PDRN-based interventions across skin rejuvenation, postoperative scar prevention, and wound healing. Although favorable outcomes were reported across all domains, treatment effects appeared to increase progressively from aesthetic rejuvenation to scar modulation and ultimately to wound repair. This pattern suggests that the clinical performance of nucleotide-based therapies may depend not only on their biological activity but also on the regenerative demands of the target tissue. Whereas aesthetic interventions primarily address chronic structural alterations associated with skin aging, wound-healing applications involve active biological processes that may be inherently more responsive to regenerative stimulation. The strongest evidence identified in this review originated from wound-healing studies. PDRN administration was associated with significant reductions in wound surface area, improved healing scores, accelerated re-epithelialization, and shorter healing times. These findings are consistent with previous evidence indicating that nucleotide-derived therapies may enhance angiogenesis, fibroblast proliferation, extracellular matrix remodeling, and collagen deposition during tissue repair [15]. The greater magnitude of benefit observed in wound-healing settings compared with aesthetic applications may reflect the presence of active inflammatory and proliferative pathways that provide a favorable biological environment for regenerative signaling. Consequently, the therapeutic impact of PDRN appears most evident in tissues undergoing active repair rather than in chronically aged skin. By comparison, studies evaluating skin rejuvenation demonstrated more modest, although consistently favorable, outcomes. Improvements were observed in wrinkle severity, skin texture, and patient satisfaction; however, effect sizes were generally smaller, and outcome assessment was considerably more heterogeneous. Unlike wound-healing endpoints, aesthetic outcomes are influenced by baseline photoaging severity, treatment technique, patient perception, and assessment methodology. Furthermore, structural alterations associated with skin aging develop gradually and may require prolonged remodeling before clinically meaningful differences become apparent. Notably, the only study that compared PDRN directly against an active comparator, microneedling combined with platelet-rich plasma, demonstrated superior wrinkle reduction in the PDRN group, suggesting that PDRN may offer meaningful biostimulatory advantages even in aesthetic settings when evaluated against established regenerative therapies. Recent reviews similarly suggest that PN-based therapies function primarily as biostimulatory agents that improve tissue quality through progressive dermal remodeling rather than immediate structural correction [16,17].

An important observation arising from the current evidence base is the tendency to discuss PN and PDRN as a unified therapeutic category despite potential biological differences between these compounds. Although both are derived from highly purified nucleotide fragments and share regenerative properties, their clinical applications within the included studies differed substantially. PDRN was predominantly investigated in wound-healing settings, where treatment effects were generally robust and objectively measurable. Conversely, PN formulations were more frequently evaluated in aesthetic dermatology and scar modulation. Contemporary literature suggests that PDRN-mediated activation of adenosine-dependent signaling pathways may contribute significantly to angiogenesis and tissue regeneration, whereas PN formulations may exert greater influence on dermal biostimulation and extracellular matrix remodeling [5,18]. However, direct comparative studies remain limited, and current evidence does not permit definitive conclusions regarding the relative efficacy of PN and PDRN for specific clinical indications.

Interpretation of the available data should also consider several methodological characteristics of the included trials. Three rejuvenation studies employed split-face designs, which reduce interindividual variability and enhance statistical efficiency by allowing participants to serve as their own controls. However, such designs may also introduce treatment interaction and diffusion effects that can influence outcome estimates. In addition, the overall evidence base remains exploratory. Most studies enrolled relatively small cohorts, and several included fewer than 30 analyzed participants. Small studies are more susceptible to imprecise effect estimates and inflation of treatment effects, particularly when subjective outcome measures are used. Accordingly, although the direction of evidence was consistently favorable, the magnitude of benefit should be interpreted cautiously until validated by adequately powered multicenter randomized trials.

Although no serious treatment-related adverse events were reported, interpretation of safety outcomes remains constrained by small sample sizes and limited follow-up durations. Reported adverse events were generally mild, transient, and localized to treatment sites. These observations are consistent with contemporary literature describing nucleotide-based therapies as well tolerated across a range of dermatologic applications [16,18]. Nevertheless, larger prospective studies are required to better characterize uncommon adverse events and long-term safety outcomes.

To our knowledge, this is the first systematic review restricted to randomized evidence that simultaneously evaluates PN- and PDRN-based interventions across skin rejuvenation, postoperative scar prevention, and wound healing. Restriction to randomized clinical trials strengthened the overall quality of the evidence base, while prospective registration, adherence to PRISMA recommendations, and structured risk of bias assessment enhanced methodological rigor and transparency.

Several limitations should be acknowledged. The available evidence remains limited by small sample sizes, heterogeneous intervention protocols, and variability in outcome assessment. Considerable differences existed in nucleotide formulations, administration routes, treatment frequency, comparator interventions, and follow-up duration. Notably, the absence of standardized PN and PDRN preparations represents a major challenge for evidence synthesis and currently limits the identification of optimal treatment protocols. Furthermore, heterogeneity across study designs and outcome measures precluded quantitative synthesis and restricted meaningful comparison between formulations.

## Conclusions

Current randomized evidence supports the potential role of PN and PDRN therapies as regenerative interventions for skin rejuvenation, postoperative scar prevention, and wound healing. Clinical benefits were observed across all evaluated domains, with the most consistent effects reported in wound-healing applications. Overall, these therapies demonstrated favorable safety profiles and promising efficacy in enhancing tissue repair and skin quality. However, the available evidence remains limited by small sample sizes, heterogeneous treatment protocols, and variability in outcome assessment. High-quality, adequately powered randomized trials with standardized formulations and longer follow-up are required to establish optimal treatment strategies and clarify the comparative roles of PN and PDRN in dermatologic practice. 
